# Descriptive study of the role of household type and household composition on women’s reproductive health outcomes in urban Uttar Pradesh, India

**DOI:** 10.1186/1742-4755-12-4

**Published:** 2015-01-12

**Authors:** Ilene S Speizer, Peter Lance, Ravi Verma, Aimee Benson

**Affiliations:** Department of Maternal and Child Health, Gillings School of Global Public Health, The University of North Carolina at Chapel Hill, Chapel Hill, NC USA; Carolina Population Center, University of North Carolina at Chapel Hill, Chapel Hill, USA; International Center for Research on Women, Delhi, India

**Keywords:** Family planning, Institutional delivery, Household type, Mother-in-law, Urban, India

## Abstract

**Background:**

More needs to be known about the role intra-familial power dynamics play in women’s reproductive health outcomes, particularly in societies like Northern India characterized by patriarchy and extended families. The key research question we explore is: how important are living arrangements (e.g., presence of the mother-in-law, presence of an elder sister-in-law, and living in the husband’s natal home) on contraceptive use behaviors and decision to deliver at an institution?

**Methods:**

Representative data collected in 2010 from six cities in Uttar Pradesh are used to examine the above research question. This study uses multivariable logistic regression methods to examine the association between women’s household type (husband’s natal home vs. not husband’s natal home) and household composition (lives with mother-in-law; and lives with elder sister-in-law) and modern family planning use and institutional delivery.

**Results:**

More than sixty percent of women in the sample live in their husband’s natal home, one-third live with their mother-in-law, and only three percent live with an elder sister-in-law. Findings demonstrate that women who live either with the mother-in-law or in the husband’s natal home are more likely to use modern family planning than those women living neither with the mother-in-law nor in the husband’s natal home. In addition, living with an elder sister-in-law is associated with less family planning use. For institutional delivery, women who live with the mother-in-law have higher institutional delivery than those not living with the mother-in-law. Multivariable analyses demonstrate that, controlling for other factors associated with modern family planning use, women living with neither the mother-in-law nor in the husband’s natal home are the least likely to use modern family planning. Similar findings are found for institutional delivery such that those women living with neither the mother-in-law nor in the husband’s natal home are the least likely to have an institutional delivery, controlling for demographic factors associated with institutional delivery.

**Conclusions:**

Where women live and who they live with matters. Future reproductive health programs for urban India should consider these context specific factors in programs seeking to improve women’s reproductive health outcomes.

## Introduction

A key to attaining the Millennium Development Goals (MDG) generally and to reducing maternal mortality by 75% by 2015 (MDG 5) is achieving universal access to family planning (FP) and reproductive health services [1]. Gaps in attaining universal access to reproductive health and FP are most apparent among the least educated and poorest segments of the population [2–5]. Across multiple countries and in both urban and rural settings, young, poorer, and uneducated women are less likely to use modern FP and to have an institutional delivery [3–5]. These gaps in FP use and institutional delivery may be related to social barriers in contexts where mothers-in-law play an important role in household decision-making [6–8].

To date, numerous studies have been undertaken to understand barriers to modern FP use and institutional delivery globally [9–14] and in India, the site of this study [15–17]. Many of these studies examine the association between education, wealth, and decision-making autonomy and reproductive health outcomes [2, 16–20]. These studies often ignore the role of mothers-in-law and others in the household in shaping FP use and institutional delivery [17, 21–23]. Family influences are important in South Asia and particularly in Northern India, where women often move into their husband’s home after marriage, mother’s-in-law are influential in household decision-making, and FP use and institutional delivery levels are lower than neighboring areas [24–27].

Most studies to date regarding the role of living with the mother-in-law in shaping reproductive health outcomes have focused on rural areas, used qualitative data, or used nationally representative surveys [6, 8, 12, 22–24, 28]. A few exceptions are studies from urban areas in Pakistan using quantitative data that have shown that mothers-in-law remain influential, even in urban settings where women may be more empowered and mobile [7, 29, 30].

This study contributes to prior research on the role of the mother-in-law in shaping reproductive decision-making by including an additional dimension to the study. In particular, we examine the association between household type (i.e., living in the husband’s natal home or not) in combination with living with the mother-in-law and modern family planning use and institutional delivery in urban Uttar Pradesh. We hypothesize that women who live in the husband’s natal home with the mother-in-law present will have the most barriers to or influences on their reproductive health outcomes as compared to women who live in their husband’s natal home without the mother-in-law or women who live neither with the mother-in-law nor in the husband’s natal home. The key research question we explore is: how important are living arrangements (e.g., presence of the mother-in-law and an elder sister-in-law and living in the husband’s natal home) on contraceptive use behaviors and the decision to deliver at an institution?

## Methods

The data for this study were collected between January-August, 2010 in six cities in Uttar Pradesh India (Agra, Aligarh, Allahabad, Gorakhpur, Moradabad, and Varanasi). The data were collected as baseline data for the evaluation of the Urban Health Initiative, the India-based component of the Bill & Melinda Gates Foundation funded Urban Reproductive Health Initiative. At baseline, a representative sample of currently married women ages 15–49 was surveyed from each city. The goal was to survey about 3,000 women per city to permit a comprehensive evaluation of the programs at the city level. A total of 17,643 women were interviewed at baseline in the six cities. (See reference [16] for details of the sampling frame and study design.) Eligible households and women were approached by a female interviewer and asked to provide consent to be interviewed. Following consent, the interviewer administered a paper and pencil survey with the respondent. This project was approved by the Futures Group India Institutional Review Board (in-country approval) as well as by the Institutional Review Boards at The University of North Carolina at Chapel Hill and the International Center for Research on Women (ICRW).

For this analysis, the focus is on two outcomes: current use of modern FP and whether the last birth in the last three years was delivered in a health institution. These outcomes were chosen as they represent important reproductive health events that were measured in the baseline survey. For the analysis of modern FP use, women who were fecund and non-pregnant at the time of the study were included. Overall, 3,122 women who were pregnant or infecund were dropped. The analysis sample for the examination of modern family planning use is 14,521 unweighted (14,633 weighted). These women were asked whether they or their husband were currently using a method of family planning and among those who reported using a method, they were asked which method they were using. Responses from these two questions were coded into two groups: users of a modern method (e.g., female and male sterilization, condoms, intrauterine device, injections, oral contraceptive pills, emergency contraceptives, and dermal patch); and non-users or users of a traditional method.

For the analysis of institutional delivery, only women who had a birth in the last three years were included. The analysis sample for institutional delivery has an unweighted sample size of 5,611 and a weighted sample size of 5,427. These women were asked where they delivered their most recent birth. All women are coded as a) delivered in a health facility (public or private; hospital or clinic), or b) delivered at home or in the home of another person (e.g., natal home, relative’s home, etc.).

The key independent variables for this analysis relate to the household type and the household composition. In particular, information reported by the woman on location of residence is used to classify if the woman reports that she currently lives in her husband’s natal home or not. The two household composition variables of interest were: living with the mother-in-law and living with an elder sister-in-law. The household composition variables were created based on the household survey that included an inventory of all household members and each household member’s relationship to the head of the household. Age was also included permitting a determination if the sister-in-law was elder to the index woman. To address the fact that there is an overlap between living with the mother-in-law and in the husband’s natal home, for this analysis, we created a joint variable. This variable has four categories: lives in the husband’s natal home and with the mother-in-law; lives in husband’s natal home only, lives with mother-in-law only; and lives elsewhere (no mother-in-law; not husband’s natal home).

The analyses control for factors previously found to be associated with modern family planning use and institutional delivery in this setting [16, 23, 31]. The control variables include: age, education, wealth group, residential site (slum/non-slum), city of residence, caste, and religion. Wealth group was calculated based on reported household assets and environmental circumstances using principal components analysis methods as is done in many large demographic surveys and previously with these data [16]. The woman’s number of children born (parity) is also included in the model of institutional delivery as women having a first birth are more likely to deliver in a facility than all other women [32]. See Table 1 for the distribution of the control variables in the two analysis samples.

**Table 1:** **Descriptive characteristics of currently married women by study sample, Uttar Pradesh, India**

Characteristics	Fecund, non-pregnant women in FP sample		Women who had a birth in last 3 years (institutional delivery)	
	Percentage	Weighted N	Percentage	Weighted N
**Age**				
<25	16.9	2467	33.8	1832
25-29	22.2	3253	37.8	2049
30-34	22.2	3255	20.2	1097
35-49	38.7	5659	8.3	448
**Education**				
None	30.8	4511	33.4	1811
1-7 years education	12.4	1815	12.7	688
8-11 years education	22.1	3235	21.3	1155
12+ years education	34.7	5073	32.7	1772
**Wealth group**				
Poorest	21.2	3104	23.1	1256
Poor	20.4	2980	20.4	1108
Middle	20.5	2994	19.2	1041
Rich	19.4	2839	19.4	1054
Richest	18.5	2716	17.8	969
**Residential site**				
Slum	18.2	2666	19.6	1063
Non-slum	81.8	11967	80.4	4364
**Parity**				
1	24.3	3549	31.2	1693
2	27.3	3997	28.8	1565
3	19.9	2910	16.3	886
4+	28.5	4117	23.7	1284
**City**				
Agra	24.1	3520	26.6	1442
Aligarh	11.6	1701	13.8	748
Allahabad	19.2	2812	17.9	969
Gorakhpur	15.4	2258	14.1	763
Moradabad	9.2	1339	9.1	494
Varanasi	20.5	3003	18.6	1011
**Caste**				
Scheduled caste or tribe	18.4	2686	20.3	1103
Other backward caste	42.6	6228	46.6	2529
None	39.1	5719	33.1	1796
**Religion**				
Hindi	96.3	14096	96.8	5252
Muslim/other	3.7	537	3.2	176
**Household type and presence of mother-in-law**				
Lives in husband’s natal home with mother-in-law	27.9	4089	36.1	1960
Lives in husband’s natal home only	34.6	5056	29.1	1580
Lives with mother-in-law only	3.9	497	3.0	163
Not natal home and no mother-in-law	34.1	4992	31.8	1724
**Presence of elder sister-in-law**	2.8	408	3.5	192

All univariate and bivariate results presented are weighted; bivariate results are presented using F-tests to demonstrate significance. To examine the association of household type and household composition, we undertake multivariable logistic regression analyses. All multivariable analyses use the unweighted sample and control for the clustered study design by adjusting the standard errors using robust estimator commands in Stata statistical software.

## Results

In Table 1, descriptive characteristics of the study samples are presented. The sample for the family planning use analysis includes a large proportion of women who are age 30 or older. Conversely, for the recent birth sample, the majority of the women are under age 30. In both samples, about a third of the women have no education and another third have 12 or more years of education. In the weighted samples, nearly one-fifth of the sample is from slum areas. At the bottom of Table 1, we present the household type and composition variables for the two analysis samples. Nearly two-thirds of women live in their husband’s natal home (adding together the first two categories). One-third of the sample lives with the mother-in-law. Only about three percent of the women live with an elder sister-in-law in the household. Notably, in the family planning analysis sample more than half of women living in the husband’s natal home do not have a mother-in-law present: this may be correlated with older age whereby as the woman ages, her mother-in-law may no longer be alive. Conversely, in the recent birth sample, more than half of women living in the husband’s natal home also live with the mother-in-law. Finally, in both samples, about one third of women live neither with the mother-in-law nor in the husband’s natal home.

Not shown in Table 1 is the proportion of the population that lives in their husband’s natal home by slum residence. In both analysis samples, seventy-two percent of women who live in slums report living in their husband’s natal home whereas about 60 percent of women in the non-slum sites live in their husband’s natal home; this difference is significant. There was no difference in living with the mother-in-law or with an elder sister-in-law between slum and non-slum residents (not shown).

Table 2 provides the bivariate distribution of modern family planning use by the household type and composition variables in the weighted sample. Overall in this sample, 54 percent of women reported using a modern method of FP. Among women who live with their elder sister-in-law, 42 percent report currently using a modern FP method. The percentage using modern FP among women not living with an elder sister-in-law is significantly higher at 54 percent. Among women living only with their mother-in-law and women only living in the husband’s natal home, the use of modern family planning is the highest at 58–59 percent. Conversely, women living with both the mother-in-law and in the husband’s natal home are the least likely to use (49 percent) and those living with neither are also less likely to use (53 percent). Also shown in Table 2 are the percentages of women using contraception in each of the household type/composition categories among women living in slums and women living in non-slum areas. The patterns of use are similar for the slum and non-slum areas for family planning use. These descriptive findings are suggestive of important roles for sisters-in-law and household type/composition. However, these findings may be misleading if women who live both in their husband’s natal home and with their mother-in-law (or sister-in-law) are also younger, less educated, or poorer since these are all factors associated with non-use of modern FP. Multivariable analyses are needed to examine the association between household type and composition on modern FP use controlling for these key demographic factors.

**Table 2:** **Use of modern contraception among currently married, fecund, non-pregnant women from Uttar Pradesh, India (n = 14,633, weighted)**

Characteristics	Using modern method* %	Significance (F-Statistic p-value)
**All women**	53.8	
**Presence of elder sister-in-law**		0.000
No	54.1	
Yes	42.0	
**Household type and presence of mother-in-law**		0.000
Lives in husband’s natal home with mother-in-law	49.1	
Lives in husband’s natal home only	58.3	
Lives with mother-in-law only	58.8	
Not natal home and no mother-in-law	52.6	
**Slum sample:**		
**Household type and presence of mother-in-law**		0.000
Lives in husband’s natal home with mother-in-law	45.4	
Lives in husband’s natal home only	55.2	
Lives with mother-in-law only	55.5	
Not natal home and no mother-in-law	49.3	
**Non-slum sample:**		
**Household type and presence of mother-in-law**		0.000
Lives in husband’s natal home with mother-in-law	49.9	
Lives in husband’s natal home only	59.2	
Lives with mother-in-law only	59.2	
Not natal home and no mother-in-law	53.1	

*Modern methods include: female and male sterilization, condoms, intrauterine device, injections, oral contraceptive pills, emergency contraceptives, and dermal patch.

Table 3 presents the bivariate association between household type and composition and institutional delivery. Overall, about 69 percent of women in these urban sites report delivering their last birth in a health institution. Significant differences are observed by household type and composition, although not necessarily in the hypothesized direction. In particular, women who live with their elder sister-in-law, and women who live with the mother-in-law (in the husband’s natal home or not in the husband’s natal home) are all significantly more likely to have an institutional delivery than their counterparts not living in these settings. At the bottom of Table 3 we present the household type/composition and facility delivery for women living in slum and women in non-slum areas. Across all household type/composition categories, women living in slums are less likely to deliver in an institution. In both slums and non-slums, women living in the husband’s natal home and with the mother-in-law are the most likely to deliver in an institution. The slum women who live with the mother-in-law were the least likely to deliver in a facility (only about 50%) whereas there is little difference between the other two categories. Among women in non-slum areas, a greater percentage of women living with the mother-in-law only reported institutional delivery compared to the two other categories. To better understand the role of household type and composition, we need to control for key demographics that are related to institutional delivery and may also be related to living arrangement, such as having a first birth. Younger couples having their first births may be more likely to live with family and first births are more likely to be delivered in a health institution.

**Table 3:** **Institutional delivery among women who had a birth in the last three years from Uttar Pradesh, India (n = 5427, weighted)**

Characteristics	Institutional delivery %	Significance (F-Statistic p-value)
**All women**	68.9	
**Presence of elder sister-in-law**		
No	68.5	0.046
Yes	78.2	
**Household type and presence of mother-in-law**		
Lives in husband’s natal home with mother-in-law	77.0	0.000
Lives in husband’s natal home only	61.8	
Lives with mother-in-law only	74.2	
Not natal home and no mother-in-law	65.6	
**Slum sample:**		
**Household type and presence of mother-in-law**		0.005
Lives in husband’s natal home with mother-in-law	64.1	
Lives in husband’s natal home only	56.3	
Lives with mother-in-law only	50.8	
Not natal home and no mother-in-law	55.4	
**Non-slum sample:**		
**Household type and presence of mother-in-law**		0.000
Lives in husband’s natal home with mother-in-law	80.3	
Lives in husband’s natal home only	63.5	
Lives with mother-in-law only	77.1	
Not natal home and no mother-in-law	67.6	

Table 4 presents multivariable logistic regression odds ratios and 95% confidence intervals for the analysis of factors associated with modern FP use (Model 1) and institutional delivery (Model 2). Of particular interest are the variables at the bottom of the table. Model 1 demonstrates significant effects for living with an elder sister-in-law on modern family planning use. In particular, women living with an elder sister-in-law are significantly less likely to use modern family planning than women not living with an elder sister-in-law. In addition, controlling for the demographic factors, compared to women who neither live in the husband’s natal home nor live with the mother-in-law, women who live only with the mother-in-law or women who live in the husband’s natal home only are more likely to use modern family planning (OR: 1.29; 95% CI: 1.04, 1.58 and OR: 1.14; 95% CI: 1.05, 1.24, respectively). These results suggest that the bivariate patterns essentially still emerge after controlling for age and other demographic factors. No difference in modern family planning use is found between women who live in the husband’s natal home and with the mother-in-law and women living neither in the husband’s natal home nor with the mother-in-law. In models not shown, when the reference group is living in the husband’s natal home and with the mother-in-law, we find that both groups of just one scenario (either the husband’s natal home or the mother-in-law) were associated with greater probability of use with p-values of just over 0.05 (p = 0.058 and p = 0.061, respectively). All other variables in the model perform as expected given the findings in the previous literature on factors associated with modern family planning use in this setting [16].

**Table 4:** **Multivariable logistic regression odds ratios (95% confidence intervals) of use of modern contraception among currently married, fecund, non-pregnant women (Model 1) and for institutional delivery for women who had a birth in the last three years (Model 2), Uttar Pradesh, India**

Covariate	Model 1 modern use	Model 2 institutional delivery
**Age** **(Excluded: Under 25)**		
25-29	2.37*** (2.12, 2.66)	1.47*** (1.24, 1.73)
30-34	3.64*** (3.24, 4.08)	1.68*** (1.36, 2.09)
35+	3.89*** (3.48, 4.33)	1.54** (1.17, 2.03)
**Education** **(Excluded: No education)**		
1-7 years	1.42*** (1.27, 1.59)	1.87*** (1.58, 2.22)
8-11 years	1.49*** (1.35, 1.64)	2.53*** (2.15, 2.99)
12+ years	1.51*** (1.37, 1.67)	6.89*** (5.48, 8.67)
**Wealth** **(Excluded: Richest)**		
Poorest	0.89* (0.80, 0.99)	0.84+ (0.70, 1.01)
Poor	0.93 (0.84, 1.04)	1.07 (0.87, 1.32)
Medium	1.01 (0.90, 1.13)	1.06 (0.86, 1.32)
Rich	0.93 (0.83, 1.04)	0.88 (0.72, 1.08)
**Type of residence** **(Excluded: Non-slum)**		
Slum	0.96 (0.88, 1.04)	0.66*** (0.57, 0.77)
**City** **(Excluded: Agra)**		
Aligarh	0.70*** (0.61, 0.80)	0.58*** (0.45, 0.75)
Allahabad	0.86* (0.75, 1.00)	0.71* (0.54, 0.94)
Gorakhpur	0.87+ (0.78, 1.01)	0.44*** (0.34, 0.58)
Moradabad	1.13+ (1.00, 1.28)	0.44*** (0.34, 0.57)
Varanasi	1.11 (0.96,1.28)	0.75* (0.58,0.97)
**Caste** **(Excluded: scheduled caste or tribe)**		
Other backward caste	0.93 (0.84, 1.03)	1.12 (0.94, 1.35)
None	0.96 (0.86, 1.07)	1.57*** (1.28, 1.92)
**Religion** **(Excluded: Muslim/Other)**		
Hindi	1.06 (0.88, 1.27)	1.06 (0.74, 1.54)
**Parity** **(Excluded: 4+)**		
1	-	3.23*** (2.56, 4.08)
2	-	1.85*** (1.51, 2.28)
3	-	1.51*** (1.24, 1.82)
**Presence of elder sister-in-law** **(Excluded: Not living with sister-in-law)**		
Sister-in-Law in Residence	0.71*** (0.58, 0.87)	0.94 (0.65, 1.36)
**Household type and presence of mother-in-law** **(Excluded: Lives with neither)**		
Lives with mother-in-law only	1.29* (1.04,1.58)	0.98 (0.67, 1.45)
Lives in husband’s natal home only	1.14** (1.05, 1.24)	1.19* (1.02, 1.38)
Living in husband’s natal and with mother-in-law	1.05 (0.90, 1.15)	1.14 (0.97, 1.34)

Parity was not included in the family planning use model. +p ≤ 0.10; *p ≤ 0.05; **p ≤ 0.01; ***p ≤ 0.001.

Table 5 presents the results for the household type and composition variables stratified by slum and non-slum sample. In both the slum and non-slum sample, the effect of the elder sister-in-law remains the same. In the slum sample, we find that living in the husband’s natal home remains positive and significant as shown in Table 4. The effect of the mother-in-law only category is positive but does not attain significance probably due to a small number of women in this category. For the non-slum sample, the findings for modern family planning use are the same as the full sample.

**Table 5:** **Multivariable logistic regression odds ratios (95% confidence intervals) of use of modern contraception among currently married, fecund, non-pregnant women (Model 1) and for institutional delivery for women who had a birth in the last three years (Model 2), Uttar Pradesh, India**

Covariate	Model 1a – slum	Model 1b – non-slum	Model 2a – slum	Model 2b – non-slum
	Modern use	Modern use	Institutional delivery	Institutional delivery
	(n = 7360)	(n = 7161)	(n = 3016)	(n = 2595)
**Presence of elder sister-in-law** **(Excluded: Not living with sister-in-law)**				
Sister-in-law in residence	0.66** (0.49,0.88)	0.77+ (0.58,1.02)	0.84 (0.52,1.37)	1.12 (0.62,2.03)
**Household type and presence of mother-in-law** **(Excluded: Lives with neither)**				
Lives with mother-in-law only	1.23 (0.87,1.76)	1.32* (1.02,1.72)	0.79 (0.47,1.34)	1.35 (0.74,2.49)
Lives in husband’s natal home only	1.17* (1.03,1.32)	1.11+ (0.99,1.24)	1.20+ (0.99,1.46)	1.13 (0.89,1.42)
Living in husband’s natal and with mother-in-law	1.06 (0.93,1.21)	1.03 (0.91,1.17)	1.03 (0.83,1.27)	1.29* (1.01,1.66)

Parity was not included in the family planning use model. +p ≤ 0.10; *p ≤ 0.05; **p ≤ 0.01. models unweighted.

Model 2 in Table 4 provides the results of household composition and type on institutional delivery controlling for key demographic factors including parity. The multivariable model demonstrates that compared to women who live neither with the mother-in-law nor in the husband’s natal home, women living only in the husband’s natal home are significantly more likely to have an institutional delivery for their last birth (OR: 1.19; 95% CI: (1.02, 1.38)). None of the other household type/composition variables, including living with an elder sister-in-law, is associated with institutional delivery. The remaining demographic factors perform as expected; more educated women, lower parity births, and non-slum residents are more likely to have an institutional delivery than all others. Table 5 presents the results stratified by slum and non-slum sample. In the slum sample, the results are the same as the full model. As shown in Table 3, in the non-slum sample, women living both in the husband’s natal home and with the mother in law are more likely to have an institutional delivery than women living neither in the husband’s natal home nor with the mother-in-law. No significant difference is found comparing the two other classifications to the neither category in the non-slum category.

## Discussion

This study found that two-thirds of women live in their husband’s natal home and about half of these women also live with their mother-in-law. About a third of the sample lives neither with the mother-in-law nor in the husband’s natal home. The study findings illustrate that among women living with the mother-in-law and in the husband’s natal home and women living with neither the mother-in-law nor in the husband’s natal home the prevalence of modern family planning use is the lowest (49 and 53 percent, respectively). In addition, living with an elder sister-in-law was also found to be associated with lower overall family planning use. Women living with just the mother-in-law or women living only in the husband’s natal home (without the mother-in-law) are more likely to use modern FP than women living neither in the husband’s natal home nor with the mother-in-law and women living in the combined scenario. For institutional delivery, we find less association between household type and composition. Women living in the husband’s natal home (no mother-in-law) were significantly more likely to have an institutional delivery than women living neither with the mother-in-law nor in the husband’s natal home. In addition, in the non-slum sample, women living in the husband’s natal home and with the mother-in-law were the most likely to have an institutional delivery.

Our findings that show less family planning use and less institutional delivery among women living neither in the husband’s natal home nor with the mother-in-law contrast previous studies from South Asia. In particular, recent studies demonstrated that women living in multi-generational households are less likely to be using modern FP [7, 28] and to have an institutional delivery [23] as compared to women living in nuclear households (with just the husband, wife, and their unmarried children present). Furthermore, men living in multi-generational households tend to want larger family sizes than those in nuclear households [25] and some of their reproductive decisions may be influenced by the larger family network [7, 28]. Other studies from South Asia have demonstrated the importance of mothers-in-law in influencing sexual and reproductive health behaviors, including the timing of childbearing, use of FP, and the role of the daughter-in-law in the household [22, 24, 33, 34]. Notably, the influence of the mother-in-law may differ by whether the couple lives with the mother-in-law in the same household or not. For example, family planning use has been found to be lower when the mother-in-law lives with the couple as compared to when the couple lives separately from the mother-in-law [7].

A possible explanation for our counter-intuitive findings may be based on the focus on urban areas. Multi-generational households in South Asia are common in both rural and urban areas and may be even more important for the urban poor who have fewer resources to set up an independent household at the time of union formation. Notably, new urban residents may find themselves in settings that do not include other family members if they are the first to migrate to their urban area. These residents may be influenced by their former (possibly rural) roots in decision-making about reproductive health behaviors. These attitudes and desires are likely associated with less overall FP use and non-institutional delivery. Consistent with our hypothesis was that women who live both in the husband’s natal home and with the mother-in-law were less likely to use family planning than women in only one of these scenarios. These results are likely indicative of a cumulative effect of living within both scenarios for women in these urban settings. Our results also indicate that while only a small percentage of women live with an elder sister-in-law (i.e., wife of the husband’s older brother), these women demonstrated significantly lower overall modern family planning use. Whether this is indicative of greater social pressures to have children in multi-family and intergenerational households or is indicative of younger wives in multi-generational households needing to have a son to attain status, or to a specific influence of the elder sister-in-law is not possible to ascertain with the current data. Future studies of multi-generational households in urban settings would need to interview all of the women in the household and compare their fertility and FP behaviors to better understand the role of this family composition category.

Household type was also found to be associated with institutional delivery for one particular scenario: controlling for all other factors, women living only in their husband’s natal home were significantly more likely to have an institutional delivery than women living neither in the husband’s natal home nor with the mother-in-law. Furthermore, among women living in non-slum areas, those who lived both with the mother-in-law and in the husband’s natal home were the most likely to have an institutional delivery. These findings are interesting in light of earlier studies. In particular, Saikia and Singh [23], using nationally representative data from India, show that women living in joint households (with in-laws) are less likely to have an institutional delivery than women living in nuclear households (just the husband and wife with their children). Further, studies from rural areas have indicated that mothers-in-law play a key role after delivery of a (first) birth [24]. Our study focuses on major urban areas and hence may be different than national or rural samples given that our sample has high access to institutional delivery and more than two-thirds of women have any education; these factors may influence women’s reproductive health (particularly delivery) decision-making. Consistent with this, Hou and Ma [32], using data from Pakistan, find that first time pregnancies are more likely to be delivered in a health institution indicating the ability of first time mothers to overcome barriers to institutional delivery that are experienced by women having higher order pregnancies. Future studies, possibly using qualitative data collection, are needed to examine in greater depth urban women’s roles, household composition and type and how these are associated with their place of delivery and other reproductive health decision-making. One other factor that is likely also related to choice of an institutional delivery is the Government of India’s Janani Suraksha Yojana (JSY or safe motherhood) Scheme launched in 2005. JSY is a national conditional cash transfer program that offers incentives to women of low socioeconomic status to have an institutional delivery [27]. With this program in place, choice of institutional delivery may be influenced by desire for extra cash rather than influenced by cultural norms to deliver at home that traditionally come from relatives and extended family. It is notable, however, that we found that women from slums were significantly less likely to have an institutional delivery no matter the household composition/type. This is indicative of possible gaps in the JSY program in urban settings of Uttar Pradesh.

This study is not without limitations. First and foremost, the data for this study are cross-sectional and thus it is not possible to be assured of the direction of causality. For example, the mother-in-law present in the household could be a consequence of a recent birth; if the mother-in-law was not present prior to delivery, she is less likely to influence the place of delivery. Second, the determination of household composition was based on the household roster which indicates the relationship between all individuals listed in the roster and the head of that household. In some cases, elder women in the household who could be influential were present but could neither be categorized as an elder sister-in-law nor a mother-in-law; these women’s influences are not factored into the models. Notably, the question on place of residence (husband’s natal home or not) was self-reported by women; it is expected that this is a more reliable indicator than the created indicator of presence of the mother-in-law or presence of an elder sister-in-law.

## Conclusion

To conclude, where women live and who they live with matter for FP use but only the former seems to matter for institutional delivery in the six major urban areas of Uttar Pradesh included in this analysis. Given the important role of the sister-in-law and mother-in-law and that women living in poorer settings are less likely to use modern FP, outreach programs to slum/poor areas may be crucial for improving the social context for FP. Outreach programs can target women of reproductive age but should also include activities with other influential women and family members in the household and community. These programs should promote the importance of family planning use for spacing and limiting births. These same outreach programs targeting poor/slum sites could also promote institutional delivery and the JSY program given that we found that poorer/slum women were less likely to deliver in an institution. Finally, it is important to develop programs for those women who are the least attached to their urban setting (i.e., not in the husband’s natal home nor with the mother-in-law). These women were the least likely to use modern FP and had lower levels of institutional delivery compared to their counterparts living in the husband’s natal home and with the mother-in-law. These women potentially are the least trusting of outreach workers/visitors. Programs for these women may need to start by offering basic health care for the women and their children to help gain their trust and engagement of the husband/wife for future outreach for FP and delivery services. These types of targeted programs that address the social context within which women live are likely to have the biggest impacts on the Millennium Development Goals of reducing maternal mortality and achieving universal access to FP and reproductive health services for women and families in Uttar Pradesh, India.
